# Use and outcome of 1,220 primary total elbow arthroplasties from the Australian Orthopaedic Association National Joint Arthroplasty Replacement Registry 2008–2018

**DOI:** 10.1080/17453674.2019.1657342

**Published:** 2019-08-27

**Authors:** Jetske Viveen, Michel P J van den Bekerom, Job N Doornberg, Alesha Hatton, Richard Page, Koen L M Koenraadt, Christopher Wilson, Gregory I Bain, Ruurd L Jaarsma, Denise Eygendaal

**Affiliations:** a Department of Orthopedic and Trauma Surgery, College of Medicine and Public Health, Flinders University and Flinders Medical Centre, Adelaide, Australia;; b Shoulder and Elbow Unit, Department of Orthopedic Surgery, Onze Lieve Vrouwe Gasthuis, Amsterdam, The Netherlands;; c South Australian Health and Medical Research Institute (SAHMRI), Adelaide, South Australia;; d Australian Orthopedic Association National Joint Replacement Registry (AOANJRR), Adelaide, SA, Australia;; e Barwon Centre for Orthopaedic Research and Education (B-CORE), Barwon Health, St John of God Hospital and Deakin University, Geelong, Australia;; f Foundation for Orthopedic Research, Care & Education, Amphia Hospital, Breda, The Netherlands;; g Upper Limb Unit, Department of Orthopedic Surgery, Amphia Hospital, Breda, The Netherlands;; h Department of Orthopedic Surgery, Amsterdam University Medical Centers, Amsterdam The Netherlands

## Abstract

Background and purpose — The Australian Orthopaedic Association National Joint Replacement Registry (AOANJRR) was analyzed to determine trends in use of primary total elbow arthroplasty (TEA), the types of prostheses used, primary diagnoses, reasons for and types of revision, and whether the primary diagnosis or prosthesis design influenced the revision rate.

Patients and methods — During 2008–2018, 1,220 primary TEA procedures were reported of which 140 TEAs were revised. Kaplan–Meier estimates of survivorship were used to describe the time to first revision and hazard ratios (HR) from Cox proportional hazard models, adjusted for age and sex, were used to compare revision rates.

Results — The annual number of TEAs performed remained constant. The 3 most common diagnoses for primary TEA were fracture/dislocation (trauma) (36%), osteoarthritis (OA) (34%), and rheumatoid arthritis (RA) (26%). The cumulative percentage revision for all TEAs undertaken for any reason was 10%, 15%, and 19% at 3, 6, and 9 years. TEAs undertaken for OA had a higher revision rate compared with TEAs for trauma (HR = 1.8, 95% CI 1.1–3.0) and RA (HR = 2.0, CI 1.3–3.1). The Coonrad-Morrey (50%), Latitude (30%), Nexel (10%), and Discovery (9%) were the most used prosthesis designs. There was no difference in revision rates when these 4 designs were compared. The most common reasons for revision were infection (35%) and aseptic loosening (34%).

Interpretation — The indications for primary and revision TEA in Australia are similar to those reported for other registries. Revision for trauma is lower than previously reported.

Total elbow arthroplasty (TEA) designs have improved and the use of TEA has increased worldwide (Day et al. 2010). However, the procedure remains challenging and the results variable. A number of studies, including registry studies, have reported the outcomes of primary TEA. Although pain relief and improved function can be achieved in many patients, the complication and revision rates after TEA range from 20% to 62% (Reinhard et al. 2003, van der Lugt et al. 2005, Brinkman et al. 2007, Kim et al. 2011, Voloshin et al. 2011, Park et al. 2013) and are higher when compared with primary total hip arthroplasty (THA) and primary total knee arthroplasty (TKA) (Voloshin 2011). Revision rates vary depending on primary diagnosis, with in general less favorable results in TEA placed for posttraumatic sequalae (Fevang et al. 2009, Plaschke et al. 2014, Krukhaug et al. 2018). At 10 years TEA post trauma, prosthesis survival has been reported to be 60% while for RA it is reported to be 90% (Gill and Morrey 1998, Cil et al. 2008). The most common indications for revision surgery are symptomatic aseptic loosening, infection, polyethylene (PE) or bushing wear, and instability (Prkic et al. 2017, Geurts et al. 2019).

Primary TEA procedures are uncommon, with 0.5 procedures per 100,000 persons in Australia in 2018, compared with primary TKA and THA at 218 and 131 procedures per 100,000 persons per year respectively (AOANJRR 2018). Nationwide registries are a valuable resource to assess the performance of this uncommon procedure. Prevalence and outcomes in TEA can be identified in a community-based setting with a larger number of procedures available for analysis compared with most other types of studies. To date there have been published reports on TEA from 5 registries. These include the Finnish (Skytta et al. 2009), Scottish (Jenkins et al. 2013), Danish (Plaschke et al. 2014) Norwegian (Fevang et al. 2009, Krukhaug et al. 2018) and Swedish (Nestorson et al. 2018) arthroplasty registries (Table 1).

**Table 1.** Registry studies of TEA

Study	Registry (country)	No. of TEA	No. of prosthesis designs	Female (%)	Mean observation time (years)	No. of revisions	10-year survival (%)	Most common revision		Diagnosis
								reason	n (%)	
Skytta et al. (2009)	Finland	1,457	9	87	8.2	201	83	Loosening **^a^**	95 (47)	RA
Jenkins et al. (2013)	Scotland	1,146	NR	74	NR	140	90	Infection	86 (61)	RA, OA, trauma
Plaschke et al. (2014)	Denmark	324	7	82	8.8	68	81	Loosening **^a^**	39 (57)	RA, OA, trauma
Fevang et al. (2009)	Norway	562	9	80	6^b^	58	85	Loosening **^a^**	19 (33)	RA, OA, trauma
Krukhaug et al. (2018)	Norway	838	13	78	9^b^	158	81	Loosening **^a^**	66 (42)	RA, OA, fracture **^c^**
Nestorson et al. (2018)	Sweden	406	7	90	6	18	90	Loosening **^a^**	7 (39)	Trauma

a Aseptic loosening

b Median.

c Fracture sequelae and acute fracture

NR = not reported. RA = rheumatoid arthritis. OA = osteoarthritis.

This study reports the use and outcomes of primary TEA from the Australian Orthopaedic Association National Joint Replacement Registry (AOANJRR) and compares these results with other reported studies including registry studies. This includes: (1) the number of primary TEAs performed per year; (2) the most common indications for primary TEA; (3) the reasons they were revised; (4) the overall revision rate; and (5) the effect of primary diagnosis and type of prosthesis on the rate of revision.

## Patients and methods

### Australian Orthopaedic Association National Joint Replacement Registry

This study included all primary TEA procedures reported to the AOANJRR between January 1, 2008 and December 31, 2018. The AOANJRR commenced national data collection for TEA in 2007 and by 2017 94% of elbow arthroplasty procedures had been reported to the registry (AOANJRR 2018). Registry data are validated against health department recorded data through a sequential multi-level matching process. A matching program is run monthly to search for all primary and revision arthroplasty procedures recorded in the Registry that involve the same side and joint of the same patient, thus enabling each revision to be linked to the primary procedure. Data are also matched biannually with the Department of Health and Ageing’s National Death Index to obtain information on the date of death (AOANJRR 2018).

When a bilateral primary TEA was performed, each TEA was considered separately. Demographic data including patient characteristics (age, sex, and since 2012 ASA score), primary diagnosis, fixation, and type of prosthesis are reported. Fixation included cemented, hybrid, and cementless. Prosthesis design was identified by brand and classified as linked, unlinked, or convertible. First revision rates and reasons for revision were determined. The effect of primary diagnosis and prosthesis type on the rate of revision was also determined. The AOANJRR defines a revision as any reoperation of a previous TEA replacement where one or more of the prosthetic components are replaced, removed, or another component is added.

### Statistics

Kaplan–Meier estimates of survivorship were used to report the time to revision of a TEA, with censoring at the time of death or closure of the dataset at the end of December 2018. The unadjusted cumulative percentage revision (CPR), with 95% confidence intervals (CI), was calculated using unadjusted point-wise Greenwood estimates. Age and sex adjusted hazard ratios (HR) calculated from Cox proportional hazard models were used to compare the rate of revision between the groups. The assumption of proportional hazards was checked analytically for each model. If the interaction between the predictor and the log of time was statistically significant in the standard Cox model, then a time-varying model was estimated. Time points were selected based on the greatest change in hazard, weighted by a function of events. Time points were iteratively chosen until the assumption of proportionality was met and HRs were calculated for each selected time period. For the current study, if no time period was specified, the HR was calculated over the entire follow-up period. All tests were 2-tailed at 5% levels of significance. Statistical analysis was performed using SAS software version 9.4 (SAS Institute Inc., Cary, NC, USA).

### Ethics, funding, and potential conflicts of interest

Since no individual patient characteristics were available, approval by the human ethics research committee was not required. No funding for this study was received.

JV received an unrestricted Research Grant from the Marti-Keuning-Eckhardt Foundation, Amsterdam Movement Sciences, Jo Kolk Foundation, and Michael-van Vloten Foundation. JND received an unrestricted Postdoc Research Grant from the Marti-Keuning-Eckhardt Foundation. MPJB declares that the OLVG Hospital receives research support from Wright/Tornier unrelated to this study.

## Results

### Demographic characteristics

There were 1,220 primary TEAs reported to the AOANJRR during the study period of which 140 were revised. The majority were female (73%). The mean age was 70 years (female 71 years and male 69 years). ASA score was available for 630 (52%) primary TEA procedures. The majority (59%) had an ASA score of 3 or 4.

### Primary TEA prostheses

9 different types of prostheses were used (Table 2). The most common types were the Coonrad-Morrey (Zimmer, Inc., Warsaw, IN, USA) (n = 608; 50%) followed by the Latitude (Tornier, Montbonnot-Saint-Martin, France) (n = 344 linked and n = 17 unlinked; 30%), the Nexel (Zimmer, Inc., Warsaw, IN, USA; the Nexel became available in Australia only in 2013) (n = 121; 10%), and the Discovery (Biomet Inc, Warsaw, IN, USA) (n = 111; 9%) (Table 2). Of the types of TEA prostheses used, 4 were linked, 1 was a convertible, and 2 were unlinked designs. 2 implants were classified as undefined, because they were custom-made designs. These implants were excluded from further analysis on linked versus unlinked designs. Almost all procedures used a linked design (n = 1,189, 98%). Most prostheses were cemented (n = 1,119; 92%). The radial head was replaced in a small number of procedures (n = 43). All involved the Latitude prosthesis. The radial head was replaced in only 12% of procedures when this device was used.

**Table 2.** Data on 1,220 primary TEA

Factor		n (%)
Elbow class		
TEA without radial head component		1,177 (96)
TEA including radial head component		43 (4)
Prosthesis design		
Linked	Coonrad-Morrey **^a^**	608 (50)
	Latitude **^a,b^**	344 (28)
	Nexel **^a^**	121 (10)
	Discovery **^a^**	111 (9)
	Mutars **^a^**	5 (< 1)
Unlinked	Latitude **^a,b^**	17 (1)
	IBP **^a^**	1 (< 1)
	Souter Strathclyde **^a^**	7 (1)
Undefined	Comprehensive	4 (< 1)
	Custom-made/other	2 (< 1)
Fixation technique		
Cemented		1,119 (92)
Hybrid (ulnar cemented)		65 (5)
Hybrid (ulnar cementless)		32 (3)
Cementless		4 (< 1)

a Coonrad-Morrey (Zimmer, Inc., Warsaw, IN, USA), Discovery (Biomet, Inc., Warsaw, IN, USA), Nexel (Zimmer, Inc., Warsaw, IN, USA), Mutars (Implantcast GmbH, Buxtehude, Germany), Latitude (Tornier, Montbonnot-Saint-Martin, France), IBP (Biomet Inc, Warsaw, IN), Souter Strathclyde (Stryker, Rutherford, NJ, USA).

b The Latitude elbow prosthesis is a convertible design and can be placed either linked or unlinked.

**Figure 1.**
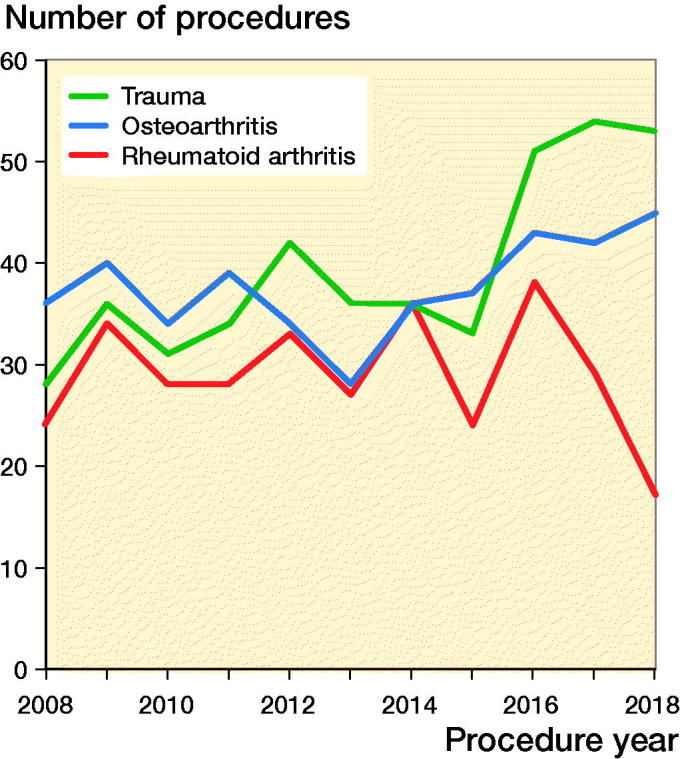
Primary total elbow replacement by primary diagnosis.

The number of primary TEAs performed each year remained constant (Table 3, see Supplementary data). The most common primary diagnoses were trauma (n = 434, 36%), OA (n = 414, 34%), and RA (n = 318, 26%). The proportion of primary TEAs undertaken for trauma has increased in recent years and is now the most common reason (Figure 1).

### Revisions of primary TEA

**Figure 2.**
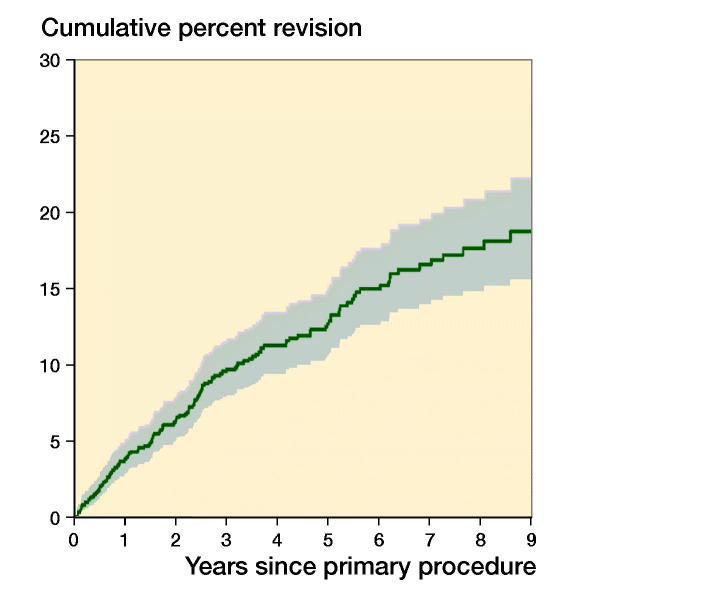
Cumulative percentage revision of primary total elbow replacement (all diagnoses).

**Figure 3.**
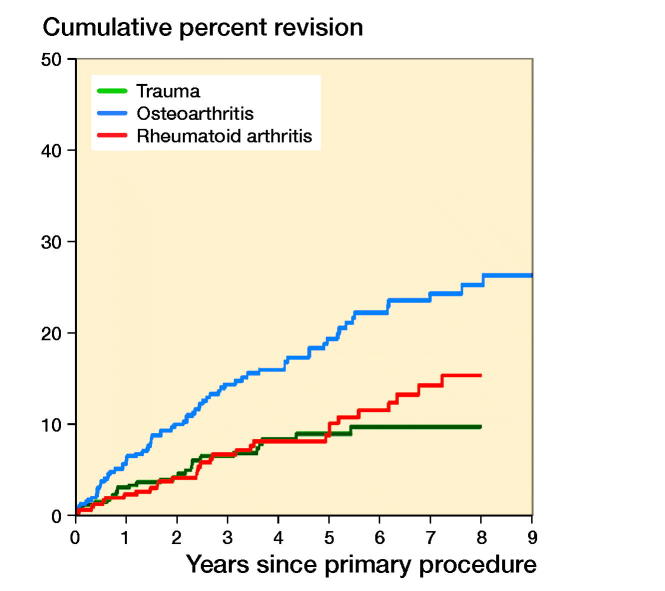
Cumulative percentage revision of primary total elbow replacement by primary diagnosis.

Of the 1,220 primary TEAs, 140 were revised. The CPR was 10%, 15%, and 19% at 3, 6, and 9 years, respectively (Table 4 and Figure 2). The revision rate varied depending on the primary diagnosis. Primary TEAs undertaken for OA were revised more frequently compared with both RA (entire period: HR = 2.0, CI 1.3–3.1) and trauma (entire period: HR = 1.8, CI 1.1–3.0). There was no statistically significant difference in the rate of revision when RA and trauma were compared (entire period: HR = 0.9, CI 0.5–1.6) (Figure 3).

**Table 4.** Yearly unadjusted cumulative percentage revision (CPR (CI)) of primary total elbow replacement (all diagnoses)

	1 year	2 years	3 years	4 years	5 years	6 years	7 years	8 years	9 years
CPR (95% CI)	4 (3–5)	7 (5–8)	10 (8–12)	11 (9–14)	13 (11–15)	15 (13–18)	17 (14–20)	18 (15–21)	19 (16–22)

**Figure 4.**
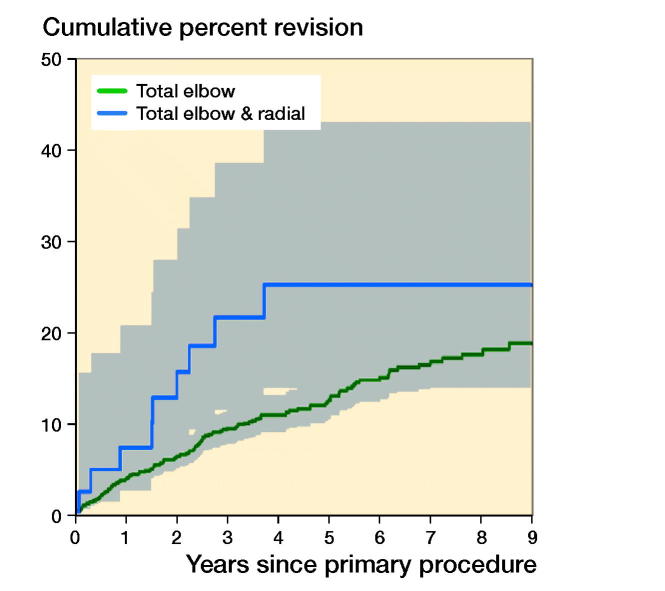
Cumulative percentage revision of primary total elbow replacement by type of primary (all diagnoses). HR adjusted for age and sex for total elbow versus total elbow & radial, entired period: HR (CI) = 1.5 (0.7–2.9). Number at risk, see below.

**Figure 5.**
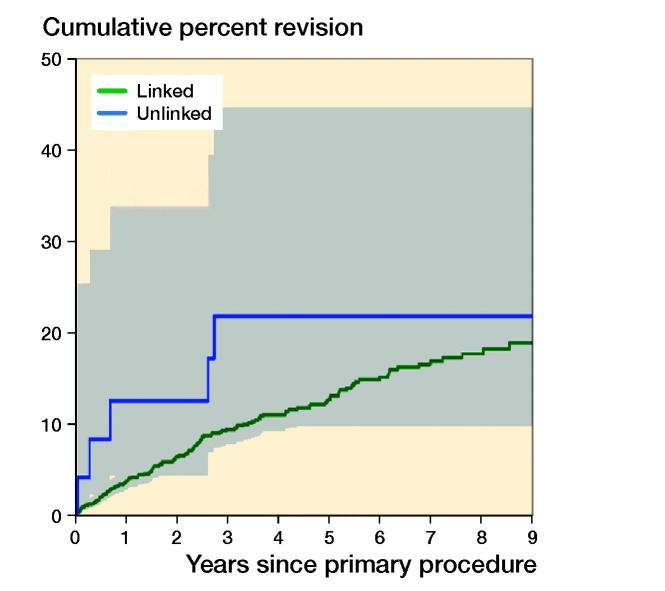
Cumulative percentage revision of primary total elbow replacement (all diagnoses). HR adjusted for age and sex for unlinked versus linked, 0–6 months: HR (CI) = 3.7 (0.9–15) and > 6 months: HR (CI) = 0.8 (0.2–2.4).

**Figure 6.**
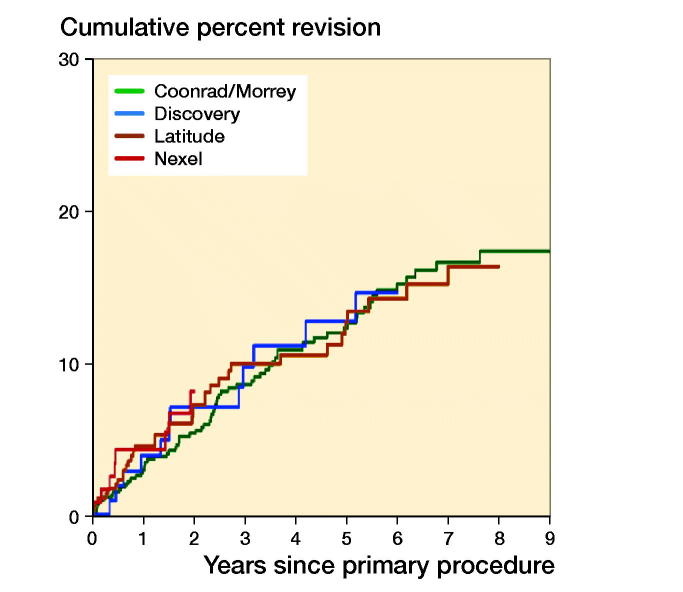
Cumulative percentage revision of primary total elbow replacement (all diagnoses). Only prostheses with over 100 procedures.

There was no statistically significant difference in the rate of revision when a radial head was used (entire period: HR = 1.5, CI 0.7–2.9) (Figure 4, see Supplementary data). There was no statistically significant difference when linked and unlinked prostheses were compared (0–6 months: HR = 3.7, CI 0.9–15.6; > 6 months: HR = 0.8, CI 0.2–2.4) (Figure 5, see Supplementary data). Revision rates were similar for the 4 most used prostheses (Coonrad Morrey, Discovery, Latitude, and Nexel, Figure 6).

The most common reasons for revision were infection (35%) and aseptic loosening (34%) (Table 5). The most common type of revision for primary TEA procedures without radial replacement undertaken for all diagnoses was of the humeral component (n = 32; 24%), followed by an elbow linking pin only (n = 25; 19%), ulnar component (n = 122; 17%), humeral/ulnar (n = 21; 16%), and cement spacer (n = 17; 13%) (Table 6, see Supplementary data). For primary TEA procedures with a radial head, the use of an ulnar component (n = 2; 22%), humeral/ulnar (n = 2; 22%), and radial head only (n = 2; 22%) were the most common types of revision (Table 6, see Supplementary data).

**Table 5.** Revision diagnosis of primary total elbow replacement by type of primary (all diagnoses). Values are frequency

Revision diagnosis	Total elbow	Total elbow and radial
Infection	46	3
Loosening	44	3
Fracture	13	
Malposition	3	
Wear bushing	3	
Implant breakage ulna	2	
Instability	1	2
Progression of disease	2	
Arthrofibrosis	1	
Implant breakage humeral	1	
Incorrect aizing	1	
Lysis	1	
Metal related pathology	1	
Prosthesis dislocation	1	1
Wear ulna	1	
Other	10	
No. revision	131	9
No. primary	1,177	43

## Discussion

This is one of the largest studies on the use and outcome of contemporary primary TEA prostheses. The annual use did not change over the 10-year period; however, there was a change in indications for primary TEA with an increased use for trauma. This has been reported previously (Gay et al. 2012). A possible explanation for this increase is that it is being used more often as a salvage procedure in selective cases of complex, comminuted, intra-articular distal humerus fractures. Its use for this diagnosis has been reported to be associated with good results (Frankle et al. 2003, McKee et al. 2009, Barco et al. 2017, Nestorson et al. 2018).

**Table:** 

Number at risk at year	0	1	2	3	4	5	6	7	8	9
Coonrad-Morrey	608	539	478	392	336	267	204	149	100	59
Discovery	111	95	83	67	55	48	46	31	22	13
Latitude	361	284	229	176	147	120	94	72	52	34
Nexel	121	96	65	37	18	8	0	0	0	0

The percentage of patients with RA is low compared with other studies with reports of up to 70% (Fevang et al. 2009, Jenkins et al. 2013, Plaschke et al. 2014, Stamp et al. 2017, Welsink et al. 2017, Krukhaug et al. 2018). The most recent Norwegian registry study identified a substantial decrease in the use of TEA for RA over the last decade (Krukhaug et al. 2018). This is likely due to the improved medical management of RA (Emery 2002, Korpela et al. 2004, Verstappen et al. 2006). The low proportion of RA patients in this study may also reflect this.

The all-cause revision rate for all diagnoses combined reported in this study is comparable to other studies (Fevang et al. 2009, Plaschke et al. 2014, Krukhaug et al. 2018). The revision rate for trauma is similar to 1 recent report (Nestorson et al. 2018). These authors considered primary TEA as a reliable treatment option for the management of complex distal humeral fractures. Although these data are supportive of that conclusion, it is our view that the use of TEA for this diagnosis, while promising, needs to be considered with some caution. This is because higher revision rates in the longer term have been reported, particularly in younger patients with posttraumatic sequelae under 65 years of age (Cil et al. 2008).

The low use of unlinked prostheses in Australia is notable. Unlinked prostheses have been popular in Europe (Fevang et al. 2009, Skytta et al. 2009, Jenkins et al. 2013, Plaschke et al. 2014, Krukhaug et al. 2018). There has, however, been an increase in the use of linked prostheses over the last decade (Krukhaug et al. 2018). Unlinked prostheses have been identified as having a higher risk of revision compared with linked designs (Plaschke et al. 2014, Geurts et al. 2019). In this study, we were unable to identify a difference between linked and unlinked prostheses because of the low use of unlinked prostheses. The main prostheses used in Australia are the Coonrad-Morrey and the Latitude (linked version). The risk of revision for these 2 devices is the same. In fact, there were similar revision rates of the 4 most commonly used prostheses, which include the Nexel and the Discovery.

The reasons for revision are similar to previous reports, with infection and aseptic loosening being the most common (Brinkman et al. 2007, Fevang et al. 2009, Plaschke et al. 2014, Prkic et al. 2017). The proportions of aseptic loosening, infection, and periprosthetic fracture in this study are comparable to most other studies (Table 1). Only Jenkins et al. (2013) reported an extremely high infection rate of 61%. However, it is uncertain whether this percentage is accurate, since no cases at all of aseptic loosening were reported in this study.

This study has several limitations. No functional or patient-reported outcomes data are available. In addition, detail on specific patient characteristics including individual comorbidities, other factors that may impact on outcome, and disease severity were not available. It was also not possible to separate acute management of trauma and later management of trauma into separate groups.

In summary, the annual use of TEA over the last decade is stable and TEA remains an uncommon procedure. The indications for primary TEA in Australia are similar to those reported by other registries. There was a trend toward the increased use of TEA for trauma and a decrease in the proportion of TEAs undertaken for RA, while the number of TEAs placed for OA remained stable. The main reasons for revision surgery (infection and aseptic loosening) and overall revision rate of 19% at 9 years are comparable to other studies as well. Primary diagnosis had a major impact on the risk of revision with procedures performed for OA having almost twice the risk compared with trauma and RA. The revision rate for TEA post trauma is lower than previously reported. The almost universal use of linked TEA designs is notable and is in contrast to the European experience.

### Supplementary data

Tables 3 and 6 and Figures 4 and 5 are available as supplementary data in the online version of this article, http://dx.doi.org/10.1080/17453674.2019.1657342

## Supplementary Material

Supplemental Material
