# Supplementary material for: Use and outcome of 1,220 primary total elbow arthroplasties from the Australian Orthopaedic Association National Joint Arthroplasty Replacement Registry 2008–2018
Source: Acta Orthop. 2019 Aug 27;90(6):511–6. doi: 10.1080/17453674.2019.1657342 (PMC6844423; doi:10.1080/17453674.2019.1657342)
Supplement: Supplemental Material [file IORT_A_1657342_SM8318.pdf]

Supplementary data

Table 3. Primary total elbow replacement by primary diagnosis. Values are frequency

Procedure year	Fracture/ dislocation (trauma)	Instability	Osteo-arthritis	Osteo-necrosis	Rheumatoid arthritis	Tumor	Other	Other inflammatory arthritides	Total
2008	28		36		24			1	89
2009	36	1	40	1	34	2	1	5	120
2010	31		34		28		2	7	102
2011	34		39		28			4	105
2012	42	1	34		33			1	111
2013	36	2	28		27			2	95
2014	36		36		36	3			111
2015	33	2	37		24	2		2	100
2016	51	1	43	1	38	1			135
2017	54		42		29	2		4	131
2018	53		45		17	3	1	2	121
Total	434	7	414	2	318	13	4	28	1,220

Table 6, Type of revision of primary total elbow replacement by type of primary (all diagnoses)

Type of revision	Total elbow	Total elbow and radial
Humeral component	32	
Elbow pin only	25	
Ulnar component	22	2
Humeral/ulnar	21	2
Cement spacer	17	1
Removal of prostheses	5	1
Minor components	3	
Ulnar cap only	3	1
Radial component	2	
Radial head only		2
Reoperation	1	
No. revision	131	9
No. primary	1,177	43

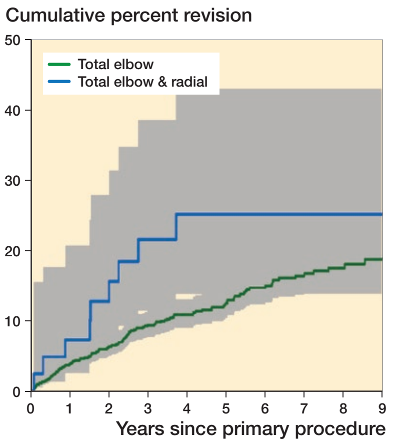

Figure 4. Cumulative percentage revision of primary total elbow replacement by type of primary (all diagnoses). HR adjusted for age and sex for total elbow versus total elbow & radial, entired period: HR (CI) = 1.5 (0.7–2.9). Number at risk, see below.

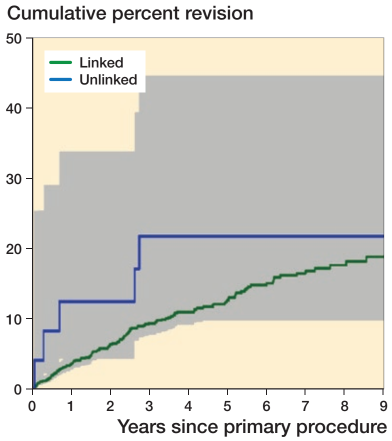

Figure 5. Cumulative percentage revision of primary total elbow replacement (all diagnoses). HR adjusted for age and sex for unlinked versus linked, 0–6 months: HR (CI) = 3.7 (0.9–15) and > 6 months: HR (CI) = 0.8 (0.2–2.4).

Number at risk at year	0	1	2	3	4	5	6	7	8	9
Total elbow	1,177	987	833	655	541	430	332	242	164	99
Total elbow & radial	43	37	30	23	20	18	17	15	13	9
